# Dl‐3‐n‐butylphthalide attenuates acute inflammatory activation in rats with spinal cord injury by inhibiting microglial TLR4/NF‐κB signalling

**DOI:** 10.1111/jcmm.13212

**Published:** 2017-08-25

**Authors:** Zili He, Yulong Zhou, Li Lin, Qingqing Wang, Sinan Khor, Yuqin Mao, Jiawei Li, Zengming Zhen, Jian Chen, Zhenzhen Gao, Fenzan Wu, Xie Zhang, Hongyu Zhang, Hua‐Zi Xu, Zhouguang Wang, Jian Xiao

**Affiliations:** ^1^ Department of Orthopaedics The Second Affiliated Hospital and Yuying Children's Hospital of Wenzhou Medical University Wenzhou Medical University Wenzhou Zhejiang China; ^2^ Molecular Pharmacology Research Center School of Pharmaceutical Science Wenzhou Medical University Wenzhou Zhejiang China; ^3^ Department of Molecular Pharmacology Albert Einstein College of Medicine Bronx NY USA; ^4^ Department of Neurosurgery Affiliated Cixi People's Hospital Wenzhou Medical University Ningbo China; ^5^ Department of Gastroenterology Ningbo Medical Treatment Center Li Hui‐li Hospital Ningbo Zhejiang China

**Keywords:** Dl‐3‐n‐butylphthalide (NBP), inflammatory, microglia, spinal cord injury

## Abstract

In this study, we examined the neuroprotective effects and anti‐inflammatory properties of Dl‐3‐n‐butylphthalide (NBP) in Sprague‐Dawley (SD) rats following traumatic spinal cord injury (SCI) as well as microglia activation and inflammatory response both *in vivo* and *in vitro*. Our results showed that NBP improved the locomotor recovery of SD rats after SCI an significantly diminished the lesion cavity area of the spinal cord, apoptotic activity in neurons, and the number of TUNEL‐positive cells at 7 days post‐injury. NBP inhibited activation of microglia, diminished the release of inflammatory mediators, and reduced the upregulation of microglial TLR4/NF‐κB expression at 1 day post‐injury. In a co‐culture system with BV‐2 cells and PC12 cells, NBP significantly reduced the cytotoxicity of BV‐2 cells following lipopolysaccharide (LPS) stimulation. In addition, NBP reduced the activation of BV‐2 cells, diminished the release of inflammatory mediators, and inhibited microglial TLR4/NF‐κB expression in BV‐2 cells. Our findings demonstrate that NBP may have neuroprotective and anti‐inflammatory properties in the treatment of SCI by inhibiting the activation of microglia *via* TLR4/NF‐κB signalling.

## Introduction

External mechanical force acting on the spinal cord can result in SCI, which is usually a lifelong disability. The primary mechanical insult acting on the spinal cord triggers activation of a series of secondary adverse effects, including disturbances in ionic homeostasis, local oedema, ischaemia, focal haemorrhage, free radicals, stress and activation of the inflammatory response 1. Inflammation has been shown to play a vital role in prolonging secondary tissue injury after SCI and leads to progressive deterioration of ventral horn motor neurons (VMNs) in the spinal cord 2. Anti‐inflammatory treatment has been shown to improve SCI 3, but much of the controversy still remains.

Toll‐like receptors (TLRs) are a family of innate immune receptors that are abundantly expressed on glial cells and neurons. They play an important role in mediating tissue survival and repair in the central nervous system (CNS). Recent studies have proposed TLRs as possible therapeutic targets for the treatment of CNS inflammation and infection 3. Among them, TLR4 has been identified as a major player. TLR4 can be recognized and activated by a diverse set of endogenous agonists, such as macromolecular degradation products, heat shock proteins, products of proteolysis, cellular components of ruptured cells and pro‐inflammatory cytokines 4. Several lines of evidence have suggested that TLR plays an important role in initiating the spinal inflammation seen to amyotrophic lateral sclerosis (ALS), ischaemia reperfusion injury, neuropathic pain and trauma 5, 6, 7. In a study performed with a mouse model of SCI, genetic ablation of TLR4 significantly reduced histological damage to the spinal cord and improved motor function 8. Microglia are a type of glial cell responsible for the innate immune defence in the CNS. When triggered by stimuli such as pathogens related to peripheral nerve injury and direct mechanical trauma, activated microglia express high levels of TLRs 9. A wide array of TLRs on microglia can be further stimulated by secreted cytokines, which augments the inflammatory cycle and exacerbates neuronal cell death and dysfunction due to a prolonged inflammatory state. In microglia, TLR4 is the most highly expressed TLR isoform compared to other family members. The activation of TLR4 triggers nuclear translation of NF‐κB, which further upregulates pro‐inflammatory cytokine production leading to subsequent neurotoxicity.

L‐3‐n‐butylphthalide (NBP) is a chemical component extracted from the seeds of Apium graveolens Linn, also known as the Chinese celery. It has been effective in treating ischaemic stroke patients. The success of NBP in clinical settings is related to its wide range of therapeutic benefits including anti‐oxidant 10, 11, 12, anti‐apoptotic 13, 14 and anti‐inflammatory properties 15. Furthermore, previous studies in a mouse model of ALS demonstrated that oral administration of NBP significantly improved locomotive function and extended their survival period, suggesting that NBP exerted its therapeutic effects through inhibition of NF‐κB activity and attenuated TNF‐α production 16. The therapeutic role of NBP was also shown in a mouse model of LPS‐induced sepsis. The study found that inhibition of the JNK pathway and upregulation of haeme oxygenase‐1 enzymatic activities led to a markedly reduced neuroinflammatory response 17. This study aims to elucidate the mechanisms underlying the inflammatory response after SCI and the signalling pathways that mediate NBP's beneficial effects.

## Materials and methods

### Animals

Adult female SD rats (220–250 g) were purchased from the Chinese Academy of Sciences, Shanghai, China. All animals were maintained in a temperature‐controlled environment (23–25°C) with 12‐hrs light/dark cycles and free access to food and water. A animal model of SCI was established as previously described 18. Animals were randomly divided into sham control, SCI and NBP‐treated SCI groups. Prior to the surgical procedure, animals were anaesthetized (i.p. injection, 2% (w/v) pentobarbital sodium, 40 mg/kg, Solarbio Science & Technology, Beijing, China). The T9 vertebrae of rats were ascertained by an animal digital X‐ray machine (KUB Technologies Inc. Stratford, CT 06615 USA), and a laminectomy was performed. Injuries were created using a vascular clip (15 g forces, Oscar, China) for 1 m, and the incision sites were sutured. In the SCI+NBP group, NBP (dissolved in peanut oil, purity >99.5%, provided by Shijiazhuang Pharmaceutical Co., Ltd., Shijiazhuang, Hebei Province, China.) was administered by oral (80 mg/kg/day) gavage after injury and then further treated once daily until these animals were killed. In the non‐treated SCI group, an equal dose of peanut oil was administered. Animals in the sham control group were subjected to the same surgical procedure without a compression injury. After the operation, manual urinary bladder emptying was performed twice daily until the bladder function was restored in animal prior to any subsequent experiments.

### Locomotion recovery assessment

In this study, locomotor function in animals was assessed at 3, 7, 14 and 28 days following SCI. Rats were placed in an open experimental field and allowed to move freely for 5 min. Crawling ability was evaluated according to the 21‐point Basso–Beattie–Bresnahan (BBB) scale. For the inclined plane test, rats were tested in two positions (right‐side or left‐side up) on a testing apparatus (a board covered with a rubber mat containing horizontal ridges spaced 3 mm apart). For each position, the maximum angle at which a rat could retain its position for 5 sec. without falling was recorded. For footprint analysis, animals were allowed to walk across a narrow box (3 m long and 10 cm wide) with painted feet. The footprints were scanned, and digitized images were analysed.

### Cell culture

PC12 cells and BV‐2 cells were purchased from the Cell Storage Centre of Wuhan University (Wuhan, China), and maintained at 37°C in a humidified atmosphere containing 5% (v/v) CO_2_. PC12 cells were cultured in DMEM (Gibco, Invitrogen, Grand Island, NY, USA) supplemented with heat‐inactivated 10% (v/v) FBS (Gibco, Invitrogen), penicillin (final concentration, 100 U/ml) and streptomycin (final concentration, 0.1 mg/ml). BV‐2 cells were cultured in MEM (Gibco, Invitrogen) supplemented with heat‐inactivated 10% (v/v) FBS, penicillin and streptomycin.

### Transwell co‐cultures

BV‐2 cells were plated onto the upper chamber of transwell inserts (0.4 μm pore size polyester membrane pre‐coated with poly‐L‐lysine; Corning, NY, USA) at a density of 3 × 10^5^ cells/ml. The transwells were positioned approximately 2 mm above PC12 cells. LPS, NBP (30 μM), LPS plus NBP or DMSO as a solvent control were added to the media of PC12 cells for 24 hrs. At the end of each experiment, mRNA and protein were harvested.

### Haematoxylin and Eosin (H&E) staining and Nissl staining

Animals were anaesthetized and transcardially perfused with 0.9% NaCl followed by 4% paraformaldehyde (PFA) at 7 days following SCI. T7–T9 spinal cord segments near the lesion epicentre were collected and fixed in 4% PFA for 24 hrs and embedded in paraffin for transverse sectioning. Five micrometer thick sections were prepared on poly‐L‐lysine‐coated slides for subsequent histopathological examination. The sections were stained with haematoxylin and eosin for H&E staining and cresyl violet for Nissl staining, in accordance with the manufacturer's instructions. All images were captured using a Nikon ECLPSE 80i (Nikon, Tokyo, Japan). The epicentre cavity area was traced using contour mapping with IPP software on sections. Nissl‐positive cells were automatically counted in five randomly selected fields per sample and quantified using IPP software.

### Apoptosis assay

DNA fragmentation was detected *in vivo* using a one‐step TUNEL Apoptosis Assay Kit (Roche, Mannheim, Germany). After deparaffinization and rehydration, sections were pre‐treated with 20 mg/ml proteinase‐K in 10 mM Tris‐HCl for 15 min. Then, sections were incubated in 0.1% sodium citrate and 0.1% Triton X‐100 solution for 10 min. After being washed with PBS, sections were incubated with 20 μl of TUNEL reaction mixture with terminal deoxynucleotidyl transferase (TdT) for 1 hr. Nuclei were stained with DAPI. Images were captured using a Nikon ECLPSE 80i at 400× magnification, and TUNEL‐positive cells were automatically counted in five randomly selected fields in the ventral horn of the spinal cord in each sample and quantified using IPP software.

### Immunofluorescence

For *in vivo* studies, spinal cord segments 7 days after surgery were fixed and prepared accordingly. Sections were incubated in 3% H_2_O_2_ for 15 min. at room temperature following by 30 min. incubation in 5% albumin from bovine serum in PBS containing 0.1% Triton X‐100 in a 37°C oven. After blocking, sections were incubated with rabbit anti‐cleaved caspase 3 (1:400; Cell Signal Technology, Danvers, MA, USA), goat anti‐Iba‐1 (1:400; Abcam, Cambridge, MA, USA), mouse anti‐NeuN (1:400; Abcam) or mouse anti‐TLR4 (1:400; Abcam) at 4°C overnight. After primary antibody incubation, sections were washed at room temperature and were incubated with appropriate secondary antibodies for 1 hr. The nuclei were stained with DAPI 19, 20. For *in vitro* studies, cells were washed in PBS, and fixed in 4% PFA for 30 min. at room temperature following by 30‐min. incubation in 5% bovine serum albumin at 37°C. Sections were stained with primary antibodies at appropriate dilutions (rabbit anti‐cleaved caspase 3 (1:400), goat anti‐Iba‐1 (1:400), mouse anti‐TLR4 (1:400) or mouse anti‐NF‐κB (1:100; Santa Cruz Biotechnology, Santa Cruz, CA) followed by incubation with appropriate secondary antibodies 21. Images were captured using a fluorescence microscope (Nikon,Tokyo,Japan) and confocal microscopy (Nikon,Tokyo,Japan).

### Quantitation of the proportion of resting and activated microglia

Percentage of field analysis was used to provide a quantitative estimate (proportional) of changes in the activation state of microglia, as previously described 22. Images were captured by confocal microscopy. Quantitative analysis was performed by blinded observers using IPP software. For cell‐density determination, the number of Iba‐1 positive cells were counted for a predefined area of the ventral horn. Resting microglia displayed small compact somata bearing long and thin ramified processes. Activated microglia exhibited marked cellular hypertrophy and retraction of processes. Background levels of signal were subtracted, and control and experimental conditions were evaluated in identical manners.

### Western blot analysis

Protein from spinal cord tissues and PC12 cells was extracted in an NP‐40 lysis buffer. An equal amount of protein was fractionated by 11.5% SDS‐PAGE, and transferred onto PVDF membranes (Bio‐Rad Laboratories, Hercules, CA, USA). Membranes were blocked with 5% freshly prepared milk‐TBST for 90 min. at room temperature and then incubated overnight at 4°C with primary antibodies (rabbit anti‐cleaved‐caspase 3 (1:1000), goat anti‐Iba‐1 (1:1000), rabbit anti‐TNF‐α (1:1000), mouse anti‐TLR4 (1:10,000), goat anti‐IL‐6 (1:300) or mouse anti‐GAPDH (1:1000) from Santa Cruz Biotechnology; mouse anti‐NF‐κB (1:400), rabbit anti‐IκB (1:400), mouse anti‐p‐IκB (1:400) from Abcam). After being washed in TBST, membranes were incubated with appropriate secondary antibodies for 1 hr at room temperature. Proteins were detected using an enhanced chemiluminescence (ECL) kit (Bio‐Rad). Signal intensities were quantified by densitometry using Image Lab 3.0 software (Bio‐Rad). Data were normalized to total or loading controls 23, 24.

### Real‐time quantitative PCR

Total RNA was extracted from cells and tissues (50–100 mg, *n* = 5 per group) using TRIZOL (Invitrogen, Carlsbad, CA, USA) according to the manufacturer's instructions. Reverse transcription and quantitative PCR were performed using the High‐Capacity cDNA Reverse Transcription Kit (Life Technologies, Carlsbad, CA, USA). Real‐time qPCR was amplified with the 7900HT Fast Real‐Time PCR System in a 10‐μl final reaction volume using SYBR Green PCR Master Mix (Bio‐Rad). Primers for IL‐1 β, TNF‐ α, IL‐ 6 and β‐actin designed against known rat sequences and mouse sequences were as follows: rat, IL‐1β forward: 5′‐ACT CCT TAG TCC TCG GCC A‐3′, reverse: 5′‐CCA TCA GAG GCA AGG AGG AA‐3′; TNF‐α forward: 5′‐TGA TCC GCG ACG TGG AA‐3′, reverse: 5′‐ACC GCC TGG AGT TCT GGA A‐3′; IL‐6 forward: 5′‐CCA AGA GGT GAG TGC TTC CC‐3′, reverse: 5′‐CTG TTG TTC AGA CTC TCT CCC T‐3′; β‐actin forward: 5′‐CCG TGA AAA GAT GAC CCA GA‐3′, reverse: 5′‐TAC GAC CAG AGG CAT ACA G‐3′; mouse, IL‐1β forward: 5′‐ACT CCT TAG TCC TCG GCC A‐3′, reverse: 5′‐CCA TCA GAG GCA AGG AGG AA‐3′; TNF‐α forward: 5′‐TGA TCC GCG ACG TGG AA‐3′, reverse: 5′‐ACC GCC TGG AGT TCT GGA A‐3′;IL‐6 forward: 5′‐CCA AGA GGT GAG TGC TTC CC‐3′, reverse: 5′‐CTG TTG TTC AGA CTC TCT CCC T‐3′; and β‐actin forward: 5′‐CCG TGA AAA GAT GAC CCA GA‐3′, reverse:5′‐TAC GAC CAG AGG CAT ACA G‐3′. Relative levels of gene expression were calculated and normalized to β‐actin. Controls were performed by omitting reverse transcriptase, cDNA or DNA polymerase 25.

### Statistical analysis

Statistical analyses were carried out using the IBM GmbH, Ehningen, Germany. Data are expressed as mean ± S.E.M. Statistical significance was determined by Student's *t*‐test if comparing only two groups or one‐way anova followed by Dunnett's *post hoc* test if analysing more than two groups. Differences were considered to be statistically significant at *P* < 0.05.

## Results

### NBP treatment decreases motor neuron loss and improves SCI recovery

**Figure 1:**
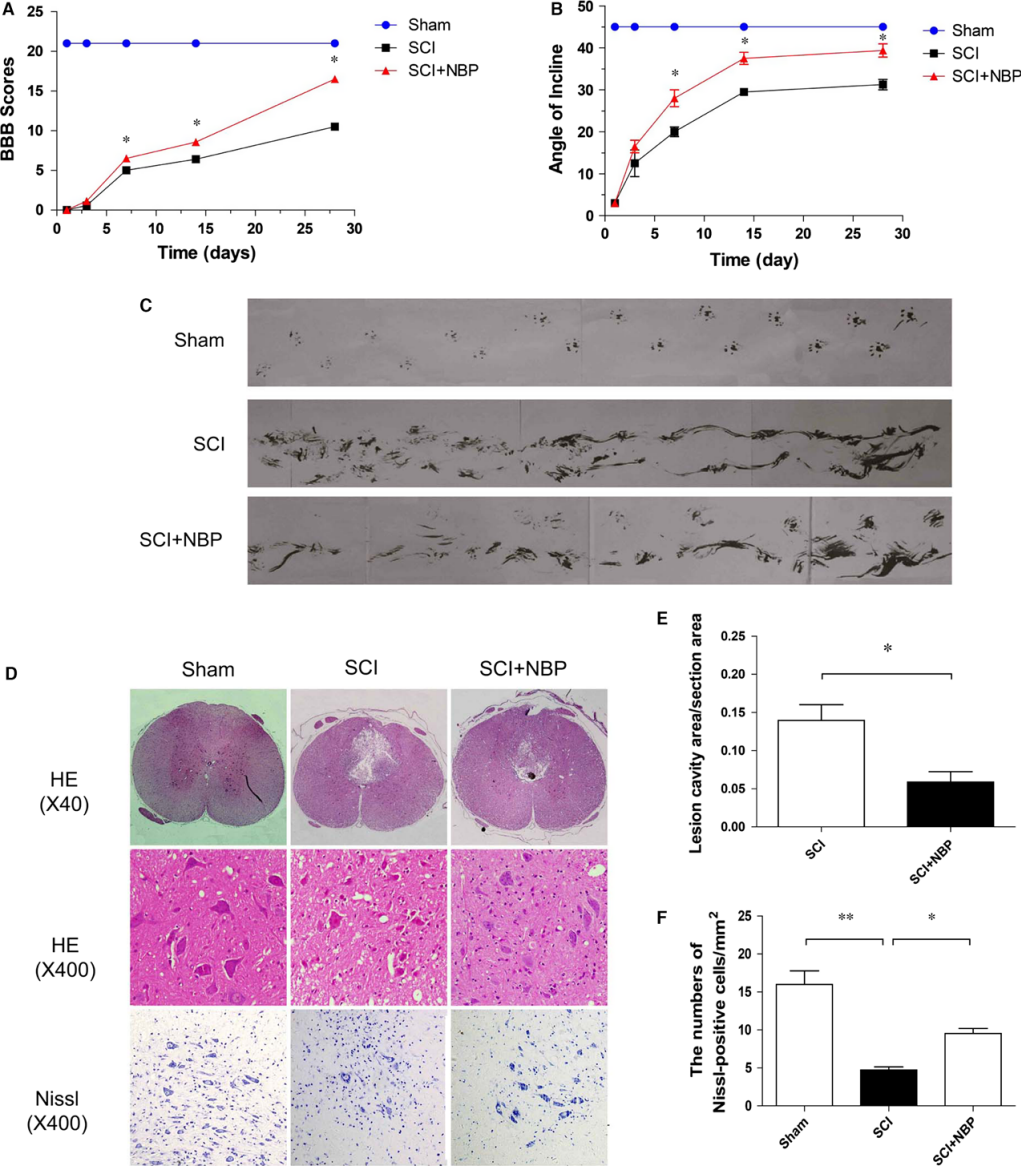
NBP increased the survival of neurons and improved SCI recovery. (**A**) The BBB scores, (**B**) the inclined plane test scores and (**C**) footprint analyses of the sham, SCI and SCI+NBP group. *P < 0.05 *versus* the SCI group, *n* = 8per group. (**D**) HE staining and Nissl staining results of the different groups at 7 days after SCI, scale bar = 1 mm and 50 μm, *n* = 5 per group. (**E**) Quantification analysis of the lesion cavity area of the SCI and NBP group at 7 days after SCI, *n* = 5 per group. (**F**) Quantification analysis of the number of Nissl staining cells at 7 days after SCI. *P < 0.05, **P < 0.01, *n* = 5 per group.

**Figure 2:**
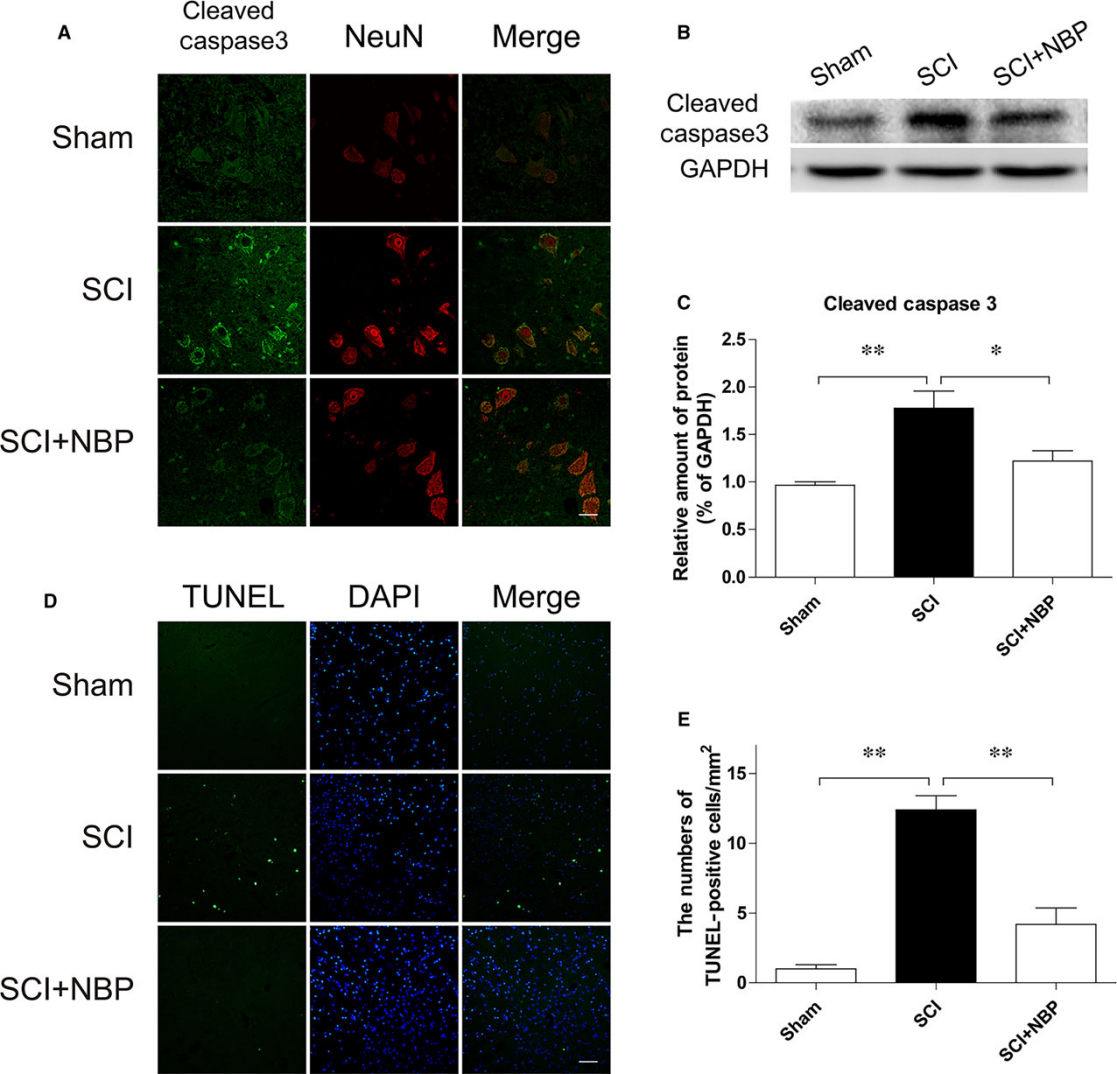
NBP attenuated apoptosis of neurons after SCI. (**A**) Double staining for NeuN‐positive neurons (red) and cleaved‐caspase 3 (green) of sections from the anterior horn of injured spinal cord in sham, SCI and SCI+NBP group at 7 days after SCI (magnification×400), scale bar = 50 μm, *n* = 5 per group. (**B** and **C**) Protein expressions and quantification of cleaved‐caspase 3 of segments from the injured spinal cord in the different groups at 7 days after SCI. *P < 0.05, **P < 0.01, *n* = 5 per group. (**D**) Double staining for TUNEL (green) and DAPI (blue) of sections from the anterior horn of injured spinal cord in the different groups at 7 days after SCI (magnification×400), scale bar = 50 μm, *n* = 5 per group. (**E**) Quantification of the number of TUNEL‐positive cells at 7 days after SCI. *P < 0.05, **P < 0.01, *n* = 5 per group.

We assessed the therapeutic effect of NBP on SCI. The degree of functional recovery was evaluated 4 weeks after injury using the 21‐point BBB rating scale, inclined plane test and footprint recordings. There was no significant difference in BBB scores between the NBP‐treated SCI and non‐treated SCI group, at 1 and 3 days post‐injury. Interestingly, the average BBB score was significantly increased in animals receiving NBP treatment when compared to the control group 7 days after injury (*P* < 0.05, Fig. 1A). The improvement was more obvious at 14 days, and the treatment group attained the highest BBB score at 28 days after injury, suggesting that NBP is an effective treatment for SCI. These findings were consistent with our observations in the inclined plane test and foot recordings in which the untreated group was outperformed by the NBP‐treated group in both experiments (Fig. 1B and C). Strikingly, the NBP‐treated rats stepped with consistent hindlimb coordination with minimal toe dragging at 28 days after injury. In contrast, the vehicle‐treated rats had inconsistent coordination with extensive toe dragging (Fig. 2C). Morphological changes in the brain were further studied by H&E staining. Progressive deterioration of the dorsal white matter and central grey matter tissue was found in the non‐treated SCI group at 7 days post‐SCI, whereas treatment of NBP significantly improved SCI pathology as evident by reduced necrosis, attenuated karyopyknosis, reduced infiltrated polymorphonuclear leucocytes and macrophages and smaller lesion cavity area (*P* < 0.05, Fig. 1D and E). Moreover, treatment of NBP markedly preserved the number of Nissl staining‐positive neurons in the treatment group as compared to the non‐treated group at 7 days after injury (*P* < 0.05, Fig. 1F). Taken together, these results suggested that NBP could effectively prevent loss of motor neurons and accelerate locomotor recovery in animals after SCI.

### NBP treatment inhibits caspase 3‐dependent neuronal cell death in rats after SCI

When compared to the non‐treated group, NBP treatment significantly reduced the amount of cleaved‐caspase 3 positive neurons and optical density at 7 days after injury (Fig. 2A). This was further supported by Western blot analyses showing reduced cleaved‐caspase 3 in the NBP‐treated group relative to their untreated counterparts (*P* < 0.05, Fig. 2B). Increased TUNEL‐positive neuronal cells in the non‐treated SCI group at 7 days after injury also indicated extensive neuronal cell death. Interestingly, treatment of NBP significantly reversed the neuronal cell death in the VMNs 1‐week post‐SCI (*P* < 0.05, Fig. 2F). Thus, these findings suggested that administration of NBP attenuated SCI‐associated neuronal cell death.

### NBP treatment prevents microglia/macrophages activation, migration and reduces pro‐inflammatory cytokine release after SCI

**Figure 3:**
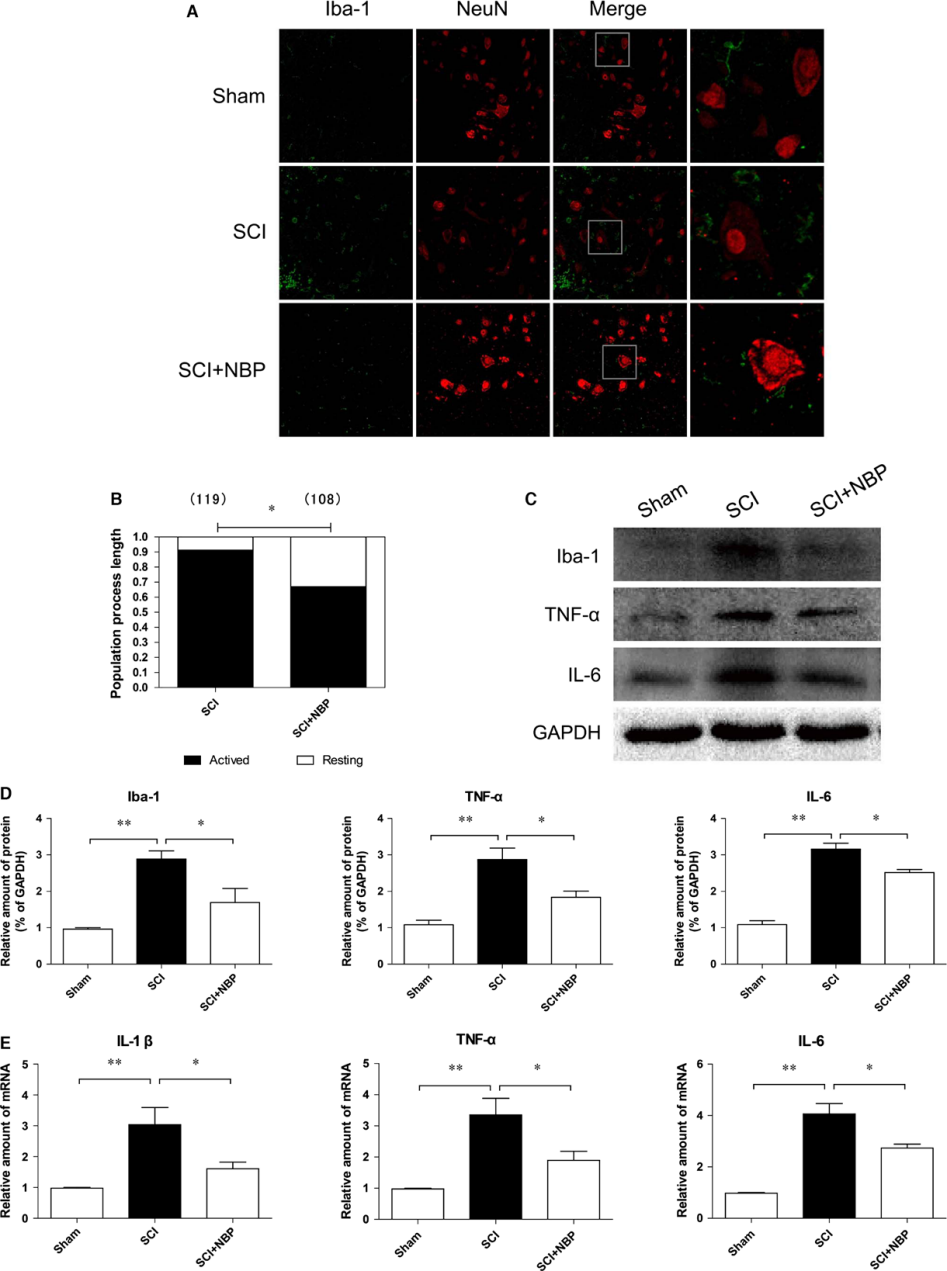
NBP prevented microglia activation, migration and reduced proinflammatory cytokines after SCI. (**A**) Double staining for NeuN‐positive neurons (red) and Iba‐1 positive microglia (green) of sections from the anterior horn of injured spinal cord in sham, SCI and SCI+NBP group (magnification× 200), scale bar = 100 μm. Right panels show high‐power views, scale bar = 50 μm, *n* = 5 per group. (**B**) Quantification of the proportion of resting and activated microglia in SCI, and SCI+NBP group. Parentheses indicate the number of microglia sampled. *P < 0.05 *versus* vehicle control, *n* = 5 per group. (**C** and **D**) Protein expressions and quantification of Iba‐1, TNF‐α and IL‐6 from segments from the injured spinal cord in the different groups at 1 day after SCI. *P < 0.05, **P < 0.01, *n* = 5 per group. (**E**) mRNA levels for IL‐1 β, TNF‐α and IL‐6 from segments of the injured spinal cord in the different groups at 1 day after SCI. *P < 0.05, **P < 0.01, *n* = 5 per group.

To determine whether NBP treatment affected microglia activation and migration, we performed immunostaining for Iba‐1 (a microglia‐specific marker) and NeuN (a neuron‐specific marker) on rat cross‐sectioned spinal tissues at 1 day after injury. In the sham group, we observed a few Iba‐1‐expressing microglia with small cell bodies and fine, ramified processes around neurons at 1 day after the sham operation. However, significantly increased Iba‐1 staining was observed in the non‐treated SCI group indicating an increased number of activated microglia with marked cellular hypertrophy and retracted processes. Treatment with NBP significantly suppressed microglial activation and migration (*P* < 0.05, Fig. 3A and B). While the sham group exhibited low levels of Iba‐1, IL‐6 and TNF‐α protein expression, NBP administration tightly regulated the production of inflammatory cytokines in microglia. Iba‐1, IL‐6 and TNF‐α protein levels were significantly reduced in the treatment group compared with their untreated counterparts after SCI (*P* < 0.01, Fig. 3C and D). Furthermore, reduced levels of Iba‐1, IL‐6 and TNF‐α mRNA were detected in the injured tissues from the NBP‐treated group as compared to the untreated group (*P* < 0.05, Fig. 3E), which further reinforced the aforementioned results that NBP treatment downregulated the production of inflammatory cytokines from microglia following SCI.

### NBP treatment suppresses microglial TLR4 expression and downstream NF‐κB signalling in rats after SCI

**Figure 4:**
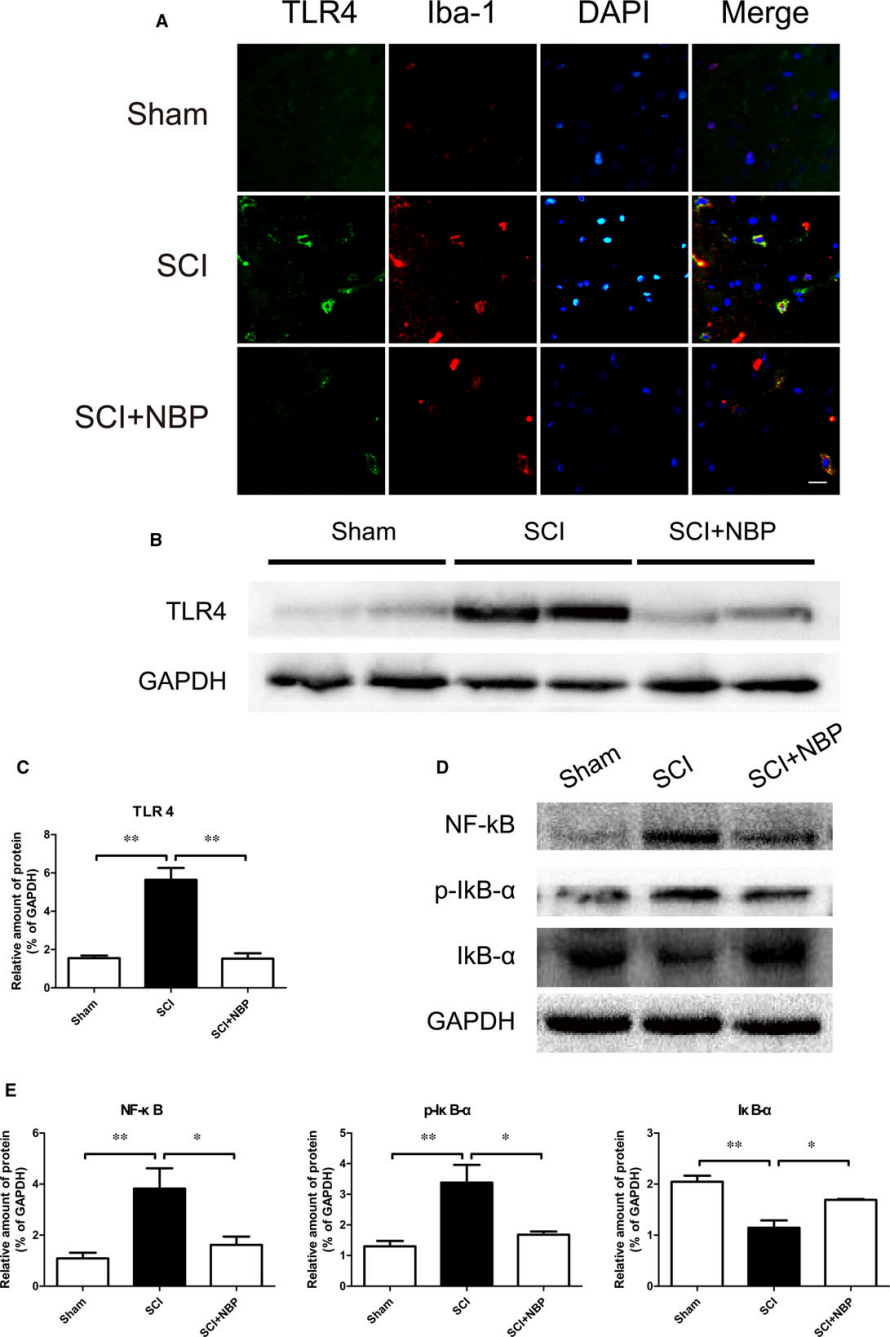
NBP suppressed microglial TLR4 expression and TLR4/NK‐κB signalling pathway protein expression after SCI. (**A**) Double staining for Iba‐1 positive microglia (red) and TLR4 (green) of sections from the anterior horn of injured spinal cord in Sham, SCI and SCI+NBP group at 1 day after SCI (magnification ×400), scale bar = 50 μm, *n* = 5 per group. (**B** ‐ **E**) Protein expressions and quantification of TLR4, NF‐κB, p‐IκB‐α and IκB‐α from segments of the injured spinal cord in the different groups at 1 day after SCI. *P < 0.05, **P < 0.01, *n* = 5 per group.

Higher co‐localization between TLR4 and Iba‐1 in microglia and increased optical density were observed in the injured tissues in the SCI group compared to sham. Nevertheless, NBP‐treated rats exhibited significantly lower TLR4‐expressing microglia as determined by double immunostaining relative to untreated controls (Fig. 4A). These observations were supported by subsequent Western blot analyses demonstrating reduced TLR4 with NBP treatment in post‐SCI animals (*P* < 0.01, Fig. 4B and C). Protein levels of NF‐κB p65 and p‐IκB‐α were also elevated in the injured tissues from the non‐treated SCI group, and reversed by NBP treatment (*P* < 0.05, Fig. 4D and E).

### NBP treatment protects neurons from LPS stimulated neurotoxicity *in vitro*

**Figure 5:**
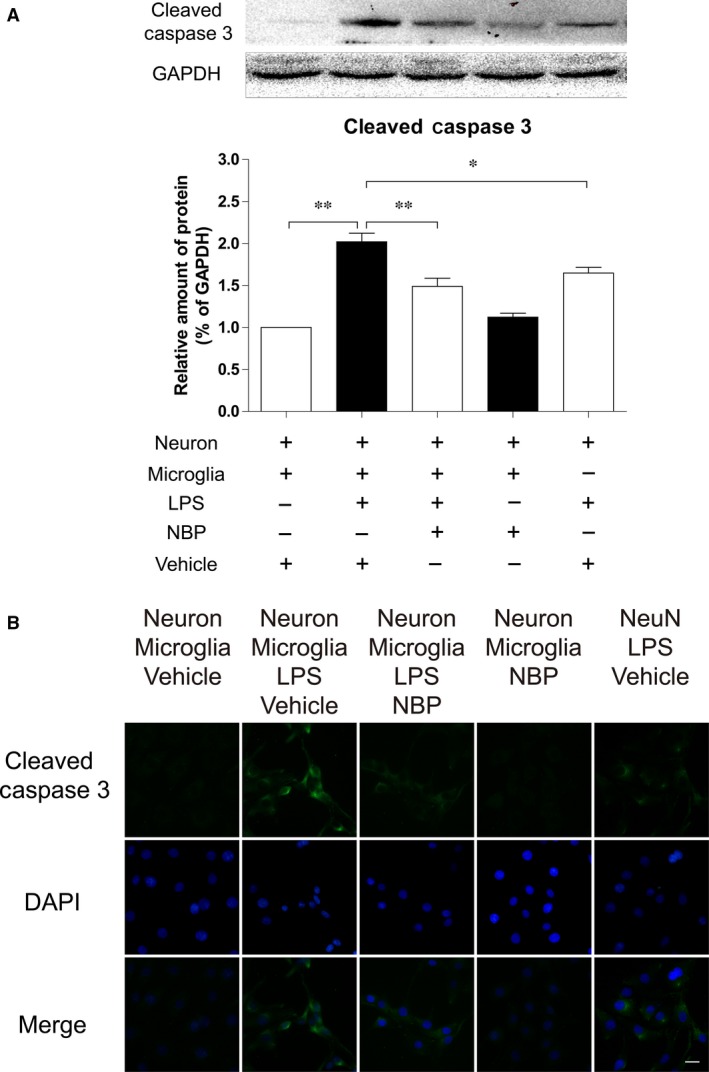
NBP reduced neuronal damage induced by LPS in co‐culture of PC12 cells and BV2 cells. (**A**) Protein expressions and quantification of cleaved‐caspase 3 in PC12 cells in co‐culture and single‐culture systems with different stimulations. *P < 0.05, **P < 0.01, *n* = 5 per group. (**B**) Double staining for cleaved‐caspase 3 (green) and DAPI (blue) in PC12 cells in co‐culture and single‐culture systems with different stimulations (magnification×400), scale bar = 50 μm, *n* = 5 per group.

We utilized a transwell co‐culture system to investigate whether NBP treatment protected neuronal cells from LPS. Significantly increased optical density was observed in cleaved‐caspase 3 positive PC12 cells when co‐treated with LPS as compared to control. Interestingly, treatment with NBP improved cell survival as shown by reduced optical density in the cleaved‐caspase 3‐expressing PC12 cells when co‐cultured with LPS. (Fig. 5A). Significantly reduced level of cleaved‐caspase 3 protein in co‐cultured PC12 cells was observed 24 hrs after LPS stimulation in the NBP treatment group as compared to the non‐treated group (*P* < 0.05, Fig. 5B and C), further supporting that the NBP treatment protected neurons from LPS‐induced neurotoxicity and subsequent cell death.

### NBP attenuates LPS‐induced microglial activation and inflammatory cytokine release *in vitro*

**Figure 6:**
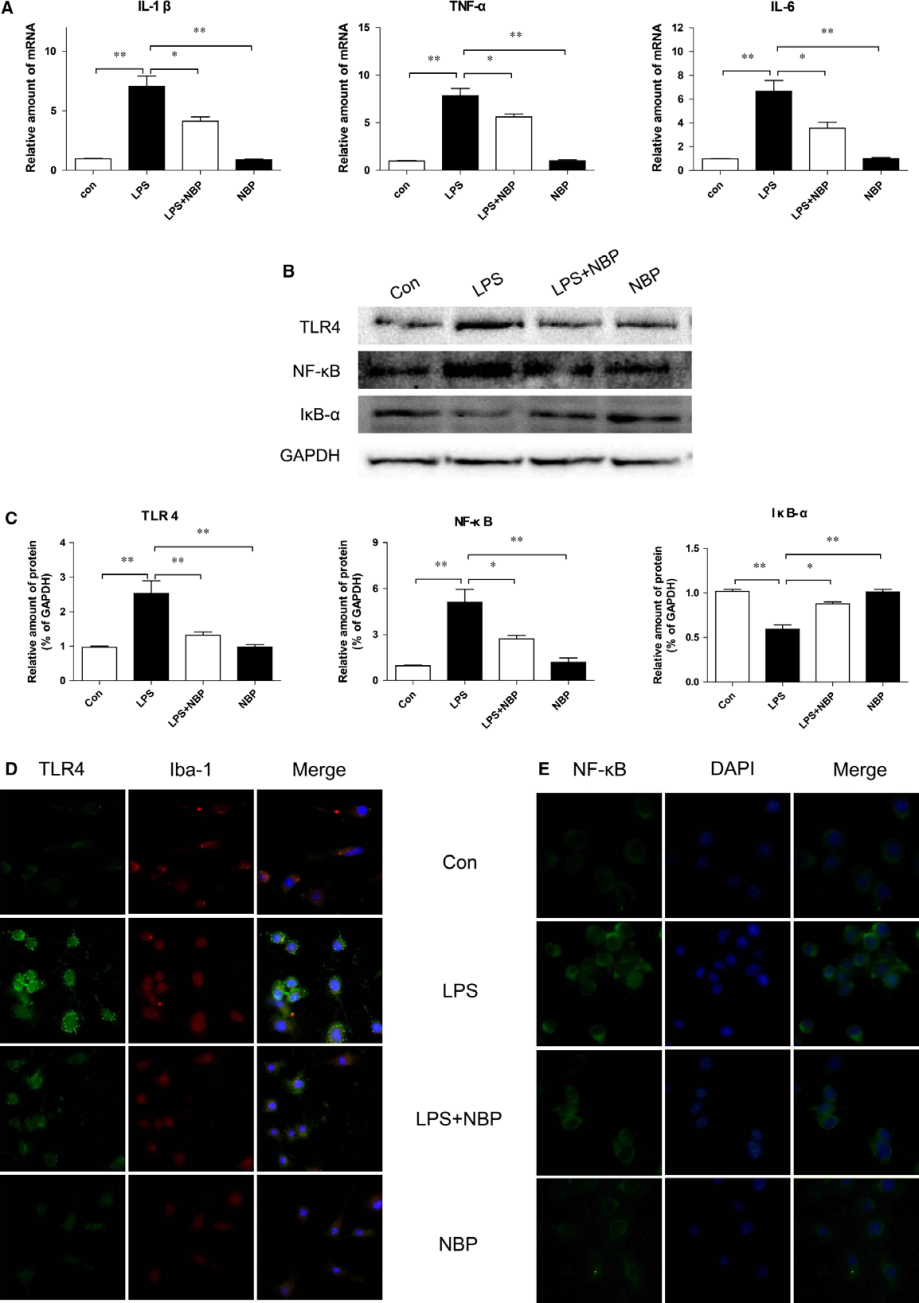
NBP attenuated the inflammatory mediator release and the expression of proteins in the microglial TLR4/MyD88/NF‐κB signalling pathway induced by LPS *in vitro*. (**A**) mRNA levels of IL‐1 β, TNF‐α and IL‐6 of BV2 cells in control, LPS, LPS+NBP and NBP groups. *P < 0.05, **P < 0.01, *n* = 5 per group. (**B** and **C**) Protein expressions and Quantification analysis of TLR4, NF‐κB and IκB‐α of BV2 cells in different groups.*P < 0.05, **P < 0.01, *n* = 5 per group. (**D**) Double staining for Iba‐1 (red) positive microglia and TLR4(green) of BV2 cells in different groups (magnification×400). (**E**) Immunofluorescence staining for NF‐kB translocation of BV2 cells in different groups (magnification×400), scale bar = 50 μm, *n* = 5 per group.

In line with our previous *in vitro* studies, we sought to examine whether treatment with NBP inhibited pro‐inflammatory cytokine production *in vitro*. It was found that LPS stimulation activated microglia with increased expression of membrane‐bound TLR4. In contrast, NBP treatment suppressed microglial activation and reduced TLR4 immunofluorescent intensity in PC12 cells (Fig. 6B). Lower levels of IL‐1β, IL‐6, and TNF‐α mRNA were found in PC12 cells when co‐treated with NBP as compared to the control group after 24 hrs of LPS simulation, indicating the production of pro‐inflammatory cytokines was also significantly reduced (*P* < 0.05, Fig. 6A).

### Treatment with NBP suppresses LPS‐induced TLR4/MyD88/NF‐κB activation *in vitro*

The effect of NBP treatment on TLR4 downstream signalling pathways in microglia was examined. Western blot analysis showed that levels of TLR4 and NF‐κB expression were markedly increased in PC12 cells after 24 hrs incubation with LPS (*P* < 0.01), whereas treatment with NBP significantly downregulated expression of them in LPS‐treated cells (*P* < 0.05). NF‐κB degradation is dependent on the presence of IκB‐a. As expected, lower expression of IκB‐a was found in PC12 cells when treated with LPS alone, however NBP treatment preserved IκB‐a protein expression after 24 hrs LPS stimulation (*P* < 0.05, Fig. 6C and D).

## Discussion

The inflammatory response is an innate immune defence mechanism that mediates destruction of foreign substances and aids the reparative phase to injury in the body. Certain specific pathological conditions can deregulate control over the inflammatory response leading to uncontrolled activation with subsequent multi‐organ failure and inevitable cell death 26, 27, 28. In SCI, the immune system has been implicated in the pathogenesis of post‐traumatic secondary injury, a delayed and progressive form of neurodegeneration that exacerbates cell death beyond the original site of trauma 29, 30, 31. After SCI, the prolonged inflammatory response potentiates resident microglial activation and proliferation, infiltration of circulating immune cells and enhanced intraspinal production of pro‐inflammatory factors. One of the adverse results related to chronic inflammatory activation is extensive neuronal cell death leading to dysfunctional behaviour 26, 27, 28. NBP is widely accepted as a prescribed medication for treating brain stroke treatment in China. In addition, several lines of evidence have shown that NBP has a diverse therapeutic effect. For instance, animal studies in a model of cerebral ischaemic showed that NBP reduced neuronal cell loss 32, 33, decreased oxidative damage 10, 11, inhibited the inflammatory response 17 and improved neuronal cell survival 13, 14. The varied beneficial effects of NBP also include improving mitochondrial function 12, promoting the proliferation, survival, differentiation of newborn neural cells and increasing neuroplasticity 32. Furthermore, treatment with NBP was shown to attenuate neuroinflammatory responses by downregulating the JNK pathway and upregulating the haeme oxygenase‐1 activity 17. In a mouse model of ALS in which locomotor function is severely impaired, mice receiving NBP displayed improved motor function due to improved neuron survival, which was mediated by inhibition of NF‐κB signalling and downstream TNF‐α production in the spinal cord 16. So far, most studies regarding NBP are focusing on its functional role in neural recovery from brain injury and neurodegenerative diseases, such as ALS. However, much less is known about the therapeutic efficacy of NBP in the treatment of SCI. In the present study, we found that NBP treatment effectively inhibited microglial activation and subsequent release of pro‐inflammatory cytokines. Mechanistically, NBP inhibiting the TLR4‐mediated NF‐κB signalling cascade, which prevented neuronal cell death. As a result, administration of NBP improved the locomotor function progressively in animals after SCI. To the best of our knowledge, this is the first study demonstrating the neuroprotective effects of NBP through inhibition of the acute neuroinflammation mediated by the TLR4/NF‐κB signalling pathway in animals with SCI.

Recent evidence supports the notion that microglia, the resident macrophages in the brain, are activated and play an important role in the inflammation. When triggered by peripheral inflammatory cues or nerve injuries, spinal microglia can be rapidly activated and respond to neurotransmitters, such as glutamate, substance P and adenosine triphosphate, released by the central terminal of primary sensory neurons. In response to the neurotransmitter release, microglia thicken their cellular processes to transform into an amoeboid form shortly and migrate towards vulnerable areas to confine the lesion. Phagocytosis plays a central role in the pathogenesis of SCI. Activated microglia release a variety of growth factors, chemokines and regulatory cytokines as well as free radicals and other toxic mediators. The secretion of these factors initiates signalling cascades, which trigger neurotoxic responses in the secondary phase of SCI, and significantly contributes to the preponderance of damage (apoptosis and necrosis) to endothelia, neurons, axons and oligodendrocytes 34, 35. Our findings are consistent with previous studies showing that microglia were rapidly activated as revealed by enhanced Iba‐1 signals and markedly increased cellular hypertrophy and retraction of processes in animals after SCI. Strikingly, NBP treatment effectively prevented the increase of microglial activation and subsequent release to inflammatory cytokines. Nevertheless, it should be noted that Iba‐1 is not a specific marker to microglia and is also expressed on monocytes/macrophages and lymphocytes. The infiltration and alteration of peripheral immune cells also have a significant impact in SCI. Consistent with the results *in vivo*, treatment with NBP significantly decreased the level of inflammatory cytokines induced by LPS stimulation. Furthermore, NBP pre‐conditioning markedly prevented cell apoptosis in PC12 cells co‐cultured with Bv‐2 (Fig. 5A and B). Taken together, these observations suggest that NBP pre‐conditioning inhibited microglial activation, reduced pro‐inflammatory cytokines release and neuronal cell death.

TLR4 is widely expressed on both CNS glia and neurons. It has been demonstrated to play an important role in initiating the cerebral inflammation and aggravating secondary injury seen after SCI in TLR4 KO mice 8. Recent studies have shown that, binding of TLR4 to endogenous or exogenous ligands (from viral or bacterial infection) activates downstream NF‐κB signalling, which enables microglial activation and induces the release of pro‐inflammatory cytokines. In agreement with previous findings, our findings have shown that increased TLR4 expression was observed in activated microglia 24 hrs after SCI, and pharmacological inhibition of TLR4 signalling attenuated levels of pro‐inflammatory mediators, including IL‐1β, IL‐6 and TNF‐α, *via* suppression of NF‐κB activity. However, there is controversy as to whether the microglial TLR4 pathway is involved during the earliest stages of SCI. Our *in vivo* findings were supported by *in vitro* observations, in which NBP treatment was able to downregulate TLR4 expression, leading to reduced IκB‐α degradation and nuclear translocation of NF‐κB transcription factors. As a result, inflammatory cytokines release was reduced thus achieving an anti‐inflammatory effect against the LPS challenge.

NBP has been approved by the State Food and Drug Administration of China and is widely used in ischaemic stroke patients. Many previous studies have demonstrated that NBP is activates a wealth of neuroprotective mechanisms including inhibition of oxidative stress 10, 11, 12, apoptosis 13, 14 and inflammation 15 as well as modulating autophagy 36, promoting neurogenesis and promoting neuroplasticity 32. In this study, we first reported that the therapeutic efficacy of NBP treatment in a rat model for SCI is due to the inhibition of inflammatory response and apoptosis. Clinical treatment strategies of NBP that involve inhibition of inflammatory activation in SCI might become possible and valuable in the future. However, the mechanisms of crosstalk between anti‐apoptotic and anti‐inflammatory signalling initiated by NBP in SCI were not clearly delineated, warranting further investigations.

Taken together, this study suggests that NBP treatment inhibits microglial activation and proliferation *via* the TLR4/NF‐κB pathway, and attenuates inflammatory responses in animals with SCI. As NBP improves the locomotor functions of these animals, this could help foment the development of effective therapeutic strategies for treating spinal cord diseases.

## Author contributions

J.X., X.H.‐Z. and Z.G.W. conceived and designed the experiments. H.Z.‐L., L.L., W.Q.‐Q., L.J.‐W., Z.Z.‐M., C.J., G.Z.‐Z., W.F.‐Z., Z.X. and Z.H.‐Y. performed the experiments. H.Z.‐L., Z.Y.‐L., X.H.‐Z. and J.X. analysed the data. KS help the manuscript edition. J.X. and Z.G.W. wrote the manuscript.

## Conflict of interest

There is no conflict of interest that could be perceived as prejudicing the impartiality of the research reported.
